# Injections of concentrated bone marrow aspirate as treatment for Discogenic pain: a retrospective analysis

**DOI:** 10.1186/s12891-020-3126-7

**Published:** 2020-02-28

**Authors:** Michael Wolff, Jon Mark Shillington, Christopher Rathbone, Shawn K. Piasecki, Brian Barnes

**Affiliations:** 1 Southwest Spine and Sports, 9913 N. 95th St, Scottsdale, AZ 85258 USA; 2 University of Texas at San Antonio, San Antonio, TX USA; 3 Isto Biologics, Hopkinton, MA USA

**Keywords:** Intradiscal, Low back pain, Platelet-rich plasma, Bone marrow aspirate, Discogenic, Mesenchymal stem cell

## Abstract

**Background:**

There are an overwhelming number of patients suffering from low back pain (LBP) resulting from disc pathology. Although several strategies are being developed pre-clinically, simple strategies to treat the large number of patients currently affected is still needed. One option is to use concentrated bone marrow aspirate (cBMA), which may be effective due to its intrinsic stem cells and growth factors.

**Methods:**

Thirty-three patients who received intradiscal injections of cBMA to relieve LBP were followed up based on Numeric Rating Scale (NRS), Oswestry Low Back Pain Disability Index (ODI), and Short Form-36 Health Survey (SF-36) scores. Patients were also subdivided into those with a pre-injection NRS > 5 and pre-injection NRS ≤ 5. The proportion of patients demonstrating at least 50% improvement (and 95% confidence intervals) from baseline at five follow-up visits for each outcome was evaluated.

**Results:**

At least 50% improvement in NRS was observed for 13.8, 45.8, 41.1, 23.5, and 38.9% of patients across five follow-up visits, out to 1 year. When stratified by high (> 5) versus low (≤ 5) baseline NRS scores, the values were 14.3, 45.5, 71.4, 22.2, and 44.4% among those with high baseline pain, and 13.3, 46.2, 20.0, 25.0, and 33.3% among those with low baseline pain. The 50% improvement rates across visits were 4.3, 28.6, 30.0, 22.2, and 30.8% for SF-36, and 4.2, 26.7, 36.4, 55.6, and 30.8% for ODI.

**Conclusions:**

Intradiscal cBMA injections may be effective to reduce pain and improve function. Patients with relatively higher initial pain may have potential for greatest improvement.

## Background

Low back pain (LBP) is among the most prevalent and costly of musculoskeletal disorders. Greater than 80% of the U.S. population has at least one episode of LBP and the associated costs due to medical expenses and lost wages can exceed $100 billion annually [1, 2]. LBP is commonly pain resulting from pathological changes to the intervertebral disc (IVD) with discogenic pain being one of the main contributors to LBP [3]. The identification of non-surgical therapies with potential to treat discogenic pain at the level of the intervertebral disc could dramatically improve patient quality of life and relieve a large national financial burden.

The IVD is comprised of an outer annular fibrosis (AF) and inner nucleus pulposus (NP). The AF is composed of concentric, dense lamellae of highly extracellular matrix-oriented type I & II collagen fibers while the NP is a less structured gelatinous extracellular matrix rich in proteoglycans (including aggrecans) and type II collagen [4–6]. Disc degeneration that occurs over time and/or subsequent to trauma is not surprising given the limited vascularity supplying the AF and NP [7]. Accordingly, a diminished capacity to cope with an inflammatory environment results in impaired IVD cell function, a situation that ultimately manifests as decreased proteoglycan synthesis and nuclear dehydration [4, 7, 8]. The untoward consequence of these events may be the development of local annular fissures, delamination, eventual internal annular disc disruption, and/or disc space narrowing. These factors may contribute to the development of low back pain that is discogenic in etiology based on a combination of the mechanical and biochemical changes within the disc. Therapies with the potential to shift the balance to a healthier IVD environment may have the ability to decrease discogenic pain [7, 9, 10].

The use of point-of-care autologous therapies, namely platelet-rich plasma (PRP) and bone marrow aspirate (BMA)-derived therapies (commonly referred to as bone marrow aspirate concentrate (BMAC), bone marrow concentrate (BMC), or concentrated bone marrow aspirate (cBMA)) have been demonstrated to be a means to decrease pain for a wide variety of orthopedic applications [11–17]. These and other preliminary cBMA studies are extremely valuable tools for the modern clinician, yet the issue of which applications are most appropriate for utilizing this technology remains. This is most likely because researchers are still uncertain of the exact detailed mechanism by which cBMA acts [18]. Many hypotheses exist, including that the results may be due to increased anti-inflammatory and anabolic cytokine and growth factor signaling contained within the physiologically buffered concentrate of BMA [18, 19]. Another theory is that the Mesenchymal Stem Cells (MSCs) found in cBMA [20] may directly mediate tissue repair, act via a paracrine pathway, or both [18, 21]. It is entirely plausible that cBMA acts by a combination of the hypothesized mechanisms mentioned, yet future studies are necessary for characterization in further detail. While continued insight into the mechanism of action is in need of further elucidation, one concern that most are in agreement with today is that injections of PRP and cBMA appear to be at least safe and reasonably effective as an interventional therapy for patients suffering from a range of orthopedic conditions [11–17, 22].

The preliminary PRP and cBMA injection studies have demonstrated promising potential to alleviating discogenic pain as a safe alternative for LBP relief [23–25]. For example, Pettine et al. reported favorable results for the use of cBMA when they demonstrated significant, prolonged reductions in pain after intradiscal injections [23, 26, 27]. Preclinical work in a chimeric rodent rotator cuff model by Nakagawa et al. suggests that these bone marrow-derived cells, in the presence of a fibrocartilage layer, have the potential to differentiate into chondrogenic cells and proliferate when transplanted in vivo [28]. Although promising, the results have yet to be confirmed in an IVD model. Nonetheless, the potential for the application of autologous therapies as a source of regenerative factors for healing within the virtually avascular IVD may be an elegant solution to an otherwise complex condition. Herein, our retrospective analysis of 33 patients adds further support for the concept of utilizing point-of-care autologous therapies to treat discogenic low back pain.

## Methods

### Patient selection for cBMA treatment

Each patient displayed objective findings of discogenic degenerative changes via MRI evidence which included disc desiccation, disc bulge or small contained protrusion (> 6 mm), and/or posterior annular tear (high intensity zone, HIZ). Patients selected as candidates for treatment also had ≥50% disc height retained compared to other lumbar discs that were considered normal. The presence of Modic changes were not considered within our patient selection. By history, patients had subjective findings consistent with chronic discogenic LBP. Pain was reported with positions and/or activities such as sitting, bending, lifting and/or transitioning from sit to stand, or maintaining a forward flexed position at the waist. Patients reported that LBP was greater than radicular symptoms (if they had any). Physical exam findings included: reports of low back pain with range of motion (forward flexion > extension), normal neurological exam (i.e., negative for weakness, sensory loss, Muscle Stretch Reflex abnormality, and/or negative SLR).

Provocative discography was performed on all patients to confirm discogenic pain and internal disc disruption. All discograms were performed by a single physician (MWW), by SIS standard guidelines [29] using a pressure manometer device and a two-needle technique with a 22-guage needle for disc entry. All patients had at least one control (i.e., normal pressure, painless) disc. Pressurization of the disc was limited to 50 psi above opening pressure or a volume of 3 ml (whichever came first). Concordant pain response was ≥6 (out of 10), described as typical pain by the patient. Volume injected into the disc, opening pressure (pressure at which contrast is seen in the disc), provocation pressure (pressure when pain is first reported), peak pressure (final pressure), character of pain response (concordant, discordant, partial concordant, no pain) was recorded for all patients. Patients were subsequently sent to obtain a post-discogram CT scan. The Modified Dallas discogram scale was used for interpretation of disc disruption on all post discogram CT scans obtained. Patients with positive concordant or partial concordant pain on discography and anatomical evidence of internal disc disruption on post-discography CT scan (Grades 1–4) were selected for treatment. No Grade 5 tears or full thickness tears were treated. All intradiscal cBMA injections were performed on a later date than the time of discography.

Study procedures were approved by the Honor Health Institutional Review Board (Scottsdale, AZ). Patients were identified by retrospective review of medical records between April 2010 and April 2015 and included those with chronic low back pain, confirmed to be discogenic in etiology. All patients had a long history of chronic LBP, typically greater than 1 year. All patients had a history of extensive conservative therapy without significant long-term benefit prior to intradiscal cBMA treatment including use of medications (NSAIDs and/or narcotics), physical therapy, and injections (epidural and/or medial branch blocks that were negative). A total of 33 patients who were treated during this time frame were reviewed (14 female, 19 male) (Table 1). During the period of observation after treatment, patients were scheduled for standard follow up appointments at 2 weeks, and 2, 3, 6, 12 months for a total of 5 visits. They were given post-injection pain medication if mutually agreed upon including NSAIDs and/or narcotics that were taken on an as-needed basis. No patients underwent other spinal interventions, such as epidurals, additional intradiscal therapies, or spinal fusion/surgery for the length of observation. There were no remarkable adverse events or complications to report.

**Table 1:** Candidate Selection Criteria for cBMA Treatment

Inclusion Criteria (*n* = 33)	
• Refractory LBP displaying discogenic degenerative changes via MRI	
• Positive concordant or partial concordant pain on discography and demonstrated internal disc disruption via CT scan (1, 2, or 3-level disc pathology accepted)	
• ≥ 50% disc height maintained at level(s) of treatment	
• No responsiveness to conservative therapy	
• Subjective findings of chronic LBP suggestive of discogenic etiology	
• Patients treated between Apr 2010 – Apr 2015	
• Patients desiring to be treated with concentrated autologous bone marrow aspirate at the level(s) of treatment	
Exclusion Criteria	
• LBP caused by any other etiologies (facet pain, stenosis, etc.)	
• < 50% disc height maintained at level(s) of treatment	
• Full-thickness tears	
• Patients who underwent any additional therapies during the follow up period	

### Bone marrow collection and processing

All procedures were performed in an ambulatory surgery center. Standard sterile technique and fluoroscopic guidance was used per protocol. Minimal to moderate conscious sedation (Versed, Fentanyl) was used as necessary to ensure patient comfort during the procedure. Local anesthesia using 2% lidocaine was used superficially and to the level of the periosteum overlying the Posterior Superior Iliac Spine (PSIS). This was completed using a 27-gauge 1.5-in. and/or 25-gauge 3.5-in. needle as necessary. An 11-gauge Jamshidi bone marrow aspiration needle was advanced through the anesthetized tissue under fluoroscopic guidance until contact was made with the periosteum of the PSIS. The needle was advanced through the cortex of the PSIS and into the trabecular bone. Similar to previously described “aspirate-rotate-aspirate” protocols [30, 31], 52 ml of BMA was drawn into a 60-ml syringe containing 8 ml anti-coagulant citrate dextrose solution (ACD-A, Isto Biologics, Hopkinton, MA) rotating the needle in 5 ml aliquots at each level, and slightly withdrawing every 20 ml. If more than one disc level was being treated then the same procedure was repeated on the contralateral side. Thereafter, 60 ml of anticoagulated BMA was placed in the Magellan Autologous Platelet Separator System (Isto Biologics, Hopkinton, MA) and spun using its Standard Cycle (2800 rpm for ~ 8 min followed by 3800 rpm for ~ 8 min) to obtain 3 to 6 ml of cBMA, containing platelets, regenerative cells including mesenchymal stem cells (MSCs), and growth factors.

### Intradiscal injections

Each target disc was identified under fluoroscopy using T12 as a reference. Superficial tissue was anesthetized to the level of the superior articular process with 1–2 ml of 1% preservative-free lidocaine. A standard, two-needle technique using an 18-gauge skin needle and a 22-gauge intradiscal needle was fluoroscopically guided in a right or left extra-pedicular approach to place the needle tip into the nucleus of the disc. Proper placement was confirmed in two planes (Anterior/Posterior and Lateral) and pictures of final placement were printed. A predetermined volume of cBMA (3 ml or less) was injected intradiscally. After cBMA injection, needles were removed carefully, and bandages and pressure were applied to the injection site(s) under sterile conditions.

### Outcome measures

Patients underwent an examination including the Oswestry Low Back Pain Disability Index (ODI) Questionnaire, Short Form-36 (SF-36) Health Survey, and Numeric Rating Scale (NRS) assessments pre-injection, and at 2, 6–8, 12, 24, and ≥ 52 weeks post-treatment.

### Statistical analysis

**Fig. 1:**
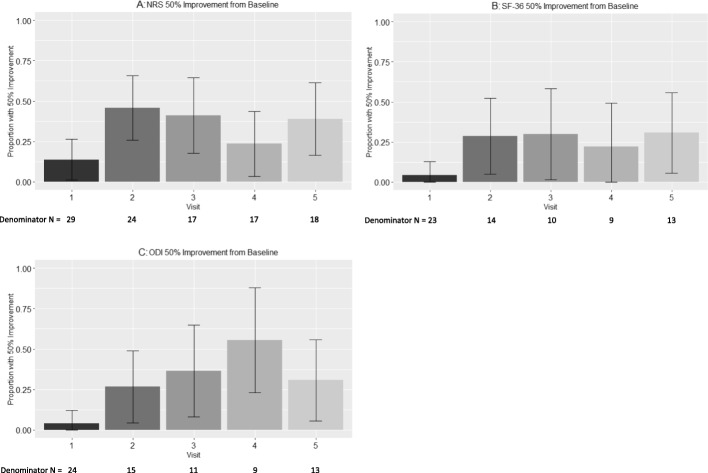
Proportion of patients with Numeric Rating Scale (NRS; **a**), Short Form-36 Health Survey (SF-36; **b**) and Oswestry Low Back Pain Disability Index (ODI; **c**) score improvements of at least 50%, and the number of patients included at each time point. Values are proportions and 95% confidence intervals. Visits are defined as follows: visit 1 (2 wks), visit 2 (6–8 wks), visit 3 (12 wks), visit 4 (6 mo), visit 5 (≥ 1 yr). The number of patients (N) with follow up data is listed below visit number

**Fig. 2:**
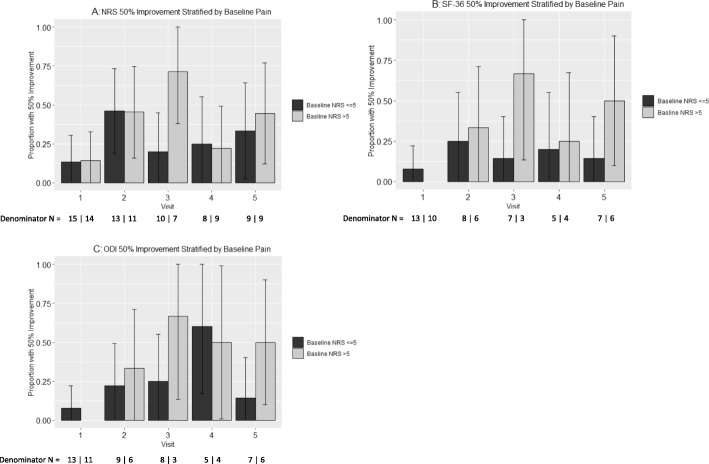
Proportion of patients with Numeric Rating Scale (NRS; **a**), Short Form-36 Health Survey (SF-36; **b**) and Oswestry Low Back Pain Disability Index (ODI; **c**) score improvements of at least 50%, and the number of patients included at each time point. Patients were subdivided into those with a pre-injection NRS ≤ 5 and those with a pre-injection NRS > 5. Values are proportions and 95% confidence intervals. Visits are defined as follows: visit 1 (2 wks), visit 2 (6–8 wks), visit 3 (12 wks), visit 4 (6 mo), visit 5 (≥ 1 yr). The number of patients (N) with follow up data is listed below visit number

SAS Version 9.4 (SAS Institute, Cary, NC) was used for all analyses. The effects of cBMA on NRS, SF-36, and ODI measures were evaluated using categorical analysis to examine the proportion of patients experiencing ≥50% change (and 95% confidence intervals) in NRS, SF-36, and ODI over time in accordance with baseline pain levels (Fig. 1). The proportion of patients experiencing ≥30% change in NRS, SF-36, and ODI was also evaluated, in addition to corresponding point reductions roughly corresponding to historically reported MCIDs (Supplementary Figures 1, 2, and 3) [32–35]. Baseline NRS was also stratified into > 5 (*n* = 15) and ≤ 5 (*n* = 18) and was incorporated into analyses depicting ≥50% change for each outcome (Fig. 2). A series of sensitivity analyses were also performed to rule out the potential impact of missing data patterns as an alternative explanation for study conclusions.

## Results

Medical records for 33 patients that were treated between April 2010 and April 2015 were reviewed which included 14 females and 19 males. Missing data patterns were consistent across patient sex. The average patient age was 45 (Table 2). Patients had a variety of pathologies related to disc disruption including: broad based disc bulges with annular tears, and small contained disc protrusions. Patients included in the analyses underwent a single treatment of intradiscal injection at 1 (*n* = 8), 2 (*n* = 16), or 3 (*n* = 9) levels (Table 2).

**Table 2:** Baseline patient characteristics and patient reported outcome scores

	Mean or N
Total # Patients	33
Age	45 (range 32–72)
Gender (Female/Male)	14/19
1 Level Injection	8
2 Level Injection	16
3 Level Injection	9
Baseline NRS	5.2 ± 0.4
Baseline SF-36	53.4 ± 2.6
Baseline ODI	36.7 ± 2.6

### Overall subjective scoring

#### NRS

The mean NRS (± S.E.M) pre-injection was 5.2 (± 0.4). The percentage of patients with at least 50% improvement in NRS scores at each post-injection time point (95% confidence intervals) was 13.8% (1.2–26.3%), 45.8% (25.9–65.8%), 41.1% (17.8–64.6%), 23.5% (3.4–43.7%), and 38.9% (16.4–61.4%) at pre-injection, and at 2, 6–8, 12, 24, and ≥ 52 weeks post-treatment, respectively (Fig. 1a). The proportion of patients with at least 30% improvement in NRS scores at each post-injection time point was also examined along with estimated MCID and is reported in Supplementary Figure 1.

#### SF-36

The mean SF-36 score (± S.E.M) pre-injection was 53.4 (± 2.6). The percentage of patients with at least 50% improvement in SF-36 scores at each post-injection time point (95% confidence intervals) was 4.3% (0.0–12.7%), 28.6% (4.9–52.2%), 30.0% (1.6–58.4%), 22.2% (0.0–49.4%), and 30.8% (5.7–55.9%) at pre-injection, and at 2, 6–8, 12, 24, and ≥ 52 weeks post-treatment, respectively (Fig. 1b). The proportion of patients with at least 30% improvement in SF-36 scores at each post-injection time point was also examined along with estimated MCID and is reported in Supplementary Figure 2.

#### ODI

The mean ODI (± S.E.M) pre-injection was 36.7 (± 2.6). The percentage of patients with at least 50% improvement in ODI scores at each post-injection time point (95% confidence intervals) was 4.2% (0.05–12.2%), 26.7% (4.3–49.0%), 36.4% (7.9–64.8%), 55.6% (23.1–88.0%), and 30.8% (5.7–55.9%) at pre-injection, and at 2, 6–8, 12, 24, and ≥ 52 weeks post-treatment, respectively (Fig. 1c). The proportion of patients with at least 30% improvement in ODI scores at each post-injection time point was also examined along with estimated MCID and is reported in Supplementary Figure 3.

### Stratified subjective scoring (baseline NRS ≤ 5 vs baseline NRS > 5)

In secondary analyses, improvement scores were examined stratified by low baseline pain (NRS ≤ 5, *n* = 18) vs. high baseline pain (NRS > 5, *n* = 15).

#### NRS

The percentage of patients with an initial NRS ≤ 5 at baseline, values were 13.3, 46.2, 20.0, 25.0, and 33.3% at pre-injection, and at 2, 6–8, 12, 24, and ≥ 52 weeks post-treatment, respectively (Fig. 2a). Among those with an initial NRS > 5 with at least 50% improvement in NRS scores at each post-injection time point was 14.3, 45.5, 71.4, 22.2, and 44.4% at pre-injection, and at 2, 6–8, 12, 24, and ≥ 52 weeks post-treatment, respectively.

#### SF-36

Improvement in SF-36 reported scores among those with an initial NRS ≤ 5, values were 7.8, 25.0, 14.3, 20.0, and 14.3%, across follow-up visits at pre-injection, and at 2, 6–8, 12, 24, and ≥ 52 weeks post-treatment. For those with an initial NRS > 5 was 0.0, 33.3, 66.7, 25.0, and 50.0% at pre-injection, and at 2, 6–8, 12, 24, and ≥ 52 weeks post-treatment, respectively (Fig. 2b).

#### ODI

Improvement in ODI among those with an initial NRS ≤ 5, values were 7.7, 22.2, 25.0, 60.0, and 14.3% across follow-up visits at pre-injection, and at 2, 6–8, 12, 24, and ≥ 52 weeks post-treatment. For those with an initial NRS > 5 was 0.0, 33.3, 66.7, 50.0, and 50.0% at pre-injection, and at 2, 6–8, 12, 24, and ≥ 52 weeks post-treatment, respectively (Fig. 2c).

## Discussion

An effective, pain-reducing, or possibly restorative treatment for chronic discogenic LBP would mark a major advancement in U.S. health care, since symptomatic management has historically been inadequate in relieving pain long-term. This 33-patient retrospective pilot study was performed to evaluate the safety and apparent effectiveness of non-surgical intradiscal injections of cBMA as a potential therapy for LBP. Based on commonly used patient reported validated outcome measures (NRS, SF-36, ODI), the major finding herein is that intradiscal injections of cBMA have the potential to reduce pain with a concomitant increase in overall patient health and function. Additionally, patients with higher baseline pain improved the most, which seems logical given that these patients had the greatest room for improvement.

This retrospective analysis has obvious limitations such as the lack of a control group, possible regression to mean, and incomplete patient data at certain time points. Thus, the interpretation of these results should be considered with prudence. Injections of concentrated Bone Marrow Aspirate were offered as a treatment option for qualified patients based upon clinical evaluation, the refractory and somewhat degenerative nature of their condition, and the relative absence of effective conservative rehabilitation strategies to avoid surgical intervention. Sub-stratification of internal disc disruption correlating to subjective response was not performed because multiple levels were treated, each with their own respective pathology. The presentation of this retrospective data was designed to report the clinical outcomes of a single physician, nonetheless it is useful to consider the similarities between these results and other larger prospective studies where cBMA and/or PRP were used to treat LBP [23–27]. For example, Tuakli-Wosurno et al. [24] observed a 35% reduction in pain from baseline to 8 weeks post-injection after injecting PRP intradiscally (NRS; 4.74 to 3.09); In the current study we observed a 40% reduction in pain during the same period in all patients (NRS; 5.2 to 3.1), and a 52% reduction in pain within the patients that had an initial NRS > 5 (NRS; 7.1 to 3.4). Similarly, Pettine et al. [23] reported a 63% reduction in pain in all subjects from baseline to 3 months post-injection with BMC (VAS; 79.3 to 29.2); In the current study we observed a 38% reduction in patient reported pain from baseline to 3 months post-injection (NRS; 5.2 to 3.2), and 62% reduction in pain within the patients that had an initial NRS score > 5 (NRS; 7.1 to 2.7). The pain reductions within our dataset were consistent in direction but varied in magnitude at later time points. Patients with baseline NRS > 5 had 32 and 38% reductions at 6 months and 1 year, respectively, whereas Pettine et al. observed 67 and 58% reductions at 6 months and 1 year, respectively [23]. Collectively, the similarities in therapeutic effectiveness are highly encouraging given that they occurred in the face of several differences among the studies (e.g., different physicians treating patients, diverse methods for product isolation, dissimilar patient populations, etc.), which could have caused widely variable results.

An interesting similarity to note is the apparent decrease in clinical effect around the 1-year mark for both this study and Pettine et al. [23]. Since each study statistically analyzed patients who only received a single cBMA injection, it is impossible to comment on whether an additional injection prior to the 1-year mark may provide a “rescue” effect. Although observational, two patients in the Pettine et al. study did choose to receive an additional injection between 6 and 12 months and both experienced additional pain relief. This topic has been fairly examined in the PRP literature, with a few high-level studies reporting that three PRP injections outperform the clinical benefit of a single PRP injection [36, 37]. However, there are obvious limitations to the interpretation of each study (i.e. different follow-up time points, different disease states, etc.) and their relevance to cBMA injections for discogenic pain. For now, it seems prudent to focus on the biological responses and clinical outcomes from a single injection protocol. Future cBMA data is thus warranted to deliver a better answer to this question. Meanwhile, the reporting of 50% patient improvement proportions (Fig. 1), stratification of initial patient pain and function status (Fig. 2), and 30% patient improvement proportions along with MCID estimates (Supplementary Figures 1, 2 and 3) from this study may help to improve potential patient benefits and expectations for these cellular therapies. The Supplementary Data reporting ≥30% improvement displays even stronger evidence for reduction in patient reported pain scoring for NRS, SF-36, and ODI.

There are several potential mechanisms that may explain these observed clinical improvements. An obvious contender would be that the addition of anabolic growth factors and regenerative stem cells to the degenerate IVD could offset its catabolic environment. Increased levels of growth factors and cytokines within PRP have been shown to improve AF and NP cell proliferation, increase glycosaminoglycan content and collagen synthesis, and stimulate gene expression for extracellular matrix proteins critical for IVD function [38, 39], even in the context of an inflammatory environment [40]. Similarly, cBMA also contains increased levels of growth factors and cytokines, yet additionally contains high levels of interleukin-1 receptor antagonist protein (IL-1ra) [20] which effectively inhibits IL-1-mediated matrix degradation [41]. It is also well known that bone marrow contains a plethora of cells that can contribute to regeneration, both directly and through paracrine effects. Mesenchymal stem cells have received the greatest amount of attention (for reviews see [9, 42]) possibly because they possess the potential for differentiation into IVD cells [43, 44]. However, their ability to increase proteoglycan content, secrete factors to reduce inflammation [45, 46], persist in the nutrient deprived IVD [47], and presence in the degenerate IVD [48] further supports the speculation that they are well-suited for treating LBP. When considering the large number of growth factors, cytokines, and regenerative cells present within cBMA it may be most appropriate to consider the way these components interact to regenerate the IVD. Further elucidation of the mechanisms involved are of great interest and important in understanding the utility of autologous therapies for LBP.

## Conclusions

The development of therapies such as autologous cBMA to treat the IVD and discogenic pain would be advantageous to treat the large number of individuals affected by this pathology. Additional studies are necessary to identify which subset of patients with discogenic LBP are most likely to experience the highest and most consistent benefits from this minimally invasive autologous therapy, and how effective this therapy is when compared to control therapies. In the future we hope to report these results, however this retrospective analysis supports the contention that autologous based therapies, including cBMA, are a logical strategy to alleviate discogenic pain and restore patient function. The use of autologous cBMA or other autologous growth factors represents a paradigm shift not just aimed at mitigating symptoms, but with the goal of providing a restorative therapy which provides long-term benefits of reduced pain and improved disc health and function.

## Supplementary information

**Additional file 1: Figure S1.** (A) Proportion and 95% confidence intervals of patients with Numeric Rating Scale (NRS) score improvements of at least 30%. Proportions at each post-injection time point were 17.2, 54.2, 52.9, 41.2, and 44.4%, respectively. (B) Estimated NRS MCID of 2 points for patients reporting at least 30% improvement in pain scores. Proportions at each post-injection time point were 17.2, 45.8, 47.1, 35.3, and 38.9%, respectively. Visits are defined as follows: visit 1 (2 wks), visit 2 (6–8 wks), visit 3 (12 wks), visit 4 (6 mo), visit 5 (≥ 1 yr). The number of patients (N) with follow up data is listed below visit number. **Figure S2.** (A) Proportion and 95% confidence intervals of patients with Short Form-36 Health Survey (SF-36) score improvements of at least 30%. Proportions at each post-injection time point were 4.4, 35.7, 30.0, 44.4, and 30.8%, respectively. (B) Estimated SF-36 MCID of 18 points for patients reporting at least 30% improvement in pain scores. Proportions at each post-injection time point were 4.4, 35.7, 30.0, 44.4, and 38.5%, respectively. Visits are defined as follows: visit 1 (2 wks), visit 2 (6–8 wks), visit 3 (12 wks), visit 4 (6 mo), visit 5 (≥1 yr). The number of patients (N) with follow up data is listed below visit number. **Figure S3.** (A) Proportion and 95% confidence intervals of patients with Oswestry Low Back Pain Disability Index (ODI) score improvements of at least 30%. Proportions at each post-injection time point were 8.3, 46.7, 45.5, 66.7, and 38.5%, respectively. (B) Estimated ODI MCID of 12 points for patients reporting at least 30% improvement in pain scores. Proportions at each post-injection time point were 8.3, 26.7, 45.5, 77.8, and 30.8%, respectively. Visits are defined as follows: visit 1 (2 wks), visit 2 (6–8 wks), visit 3 (12 wks), visit 4 (6 mo), visit 5 (≥1 yr). The number of patients (N) with follow up data is listed below visit number

**Additional file 2.** Supplementary Dataset.

## Data Availability

The datasets supporting the conclusions of this article are included within the article and its additional files.

## Abbreviations

AF: Annular fibrosis
BMAC: Bone marrow aspirate concentrate
BMC: Bone marrow concentrate
cBMA: concentrated bone marrow aspirate
IL-1: Interleukin 1
IL-1ra: Interleukin 1 receptor antagonist protein
IVD: Intervertebral disc
LPB: Low back pain
MCID: Minimal Clinically Important Difference
MSC: Mesenchymal stem cell
NP: Nucleus pulposus
NRS: Numeric Rating Scale
ODI: Oswestry Low Back Pain Disability Index
PDGF: Platelet derived growth factor
PSIS: Posterior superior iliac spine
SF-36: Short Form-36 Health Survey
TGF-β: transforming growth factor beta
VAS: Visual Analog Scale
VEGF: Vascular endothelial growth factor
