# Supplementary figures and images for: Injections of concentrated bone marrow aspirate as treatment for Discogenic pain: a retrospective analysis
Source: BMC Musculoskelet Disord. 2020 Feb 28;21:135. doi: 10.1186/s12891-020-3126-7 (PMC7049206; doi:10.1186/s12891-020-3126-7)

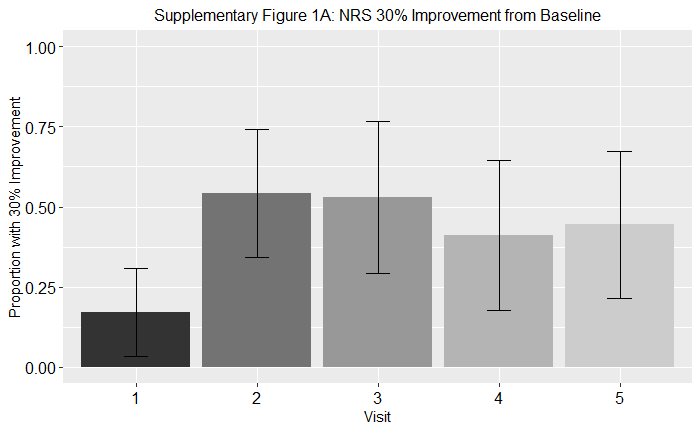

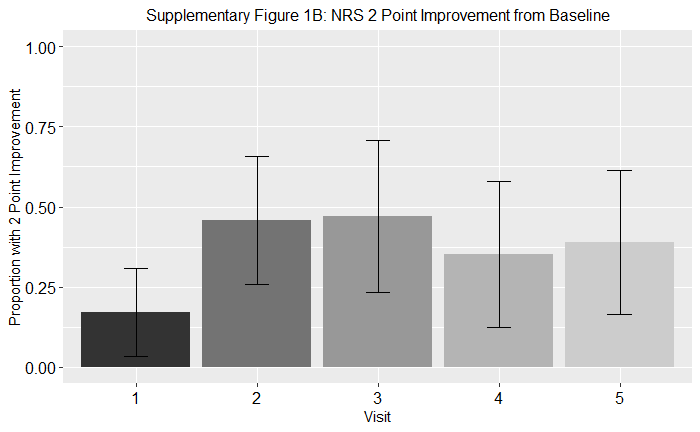


Denominator N = 29 24 17 17 18

Denominator N = 29 24 17 17 18


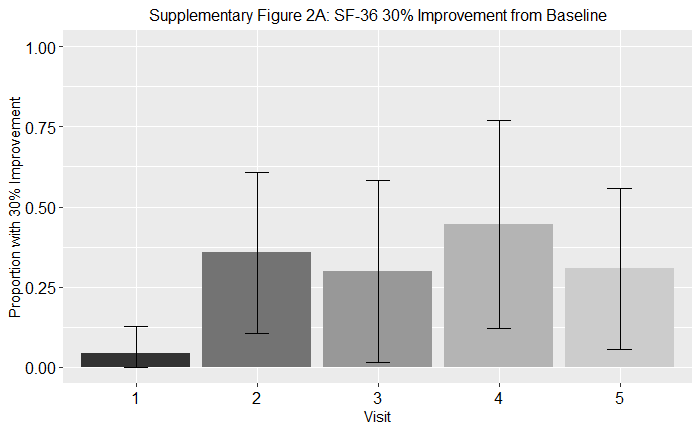

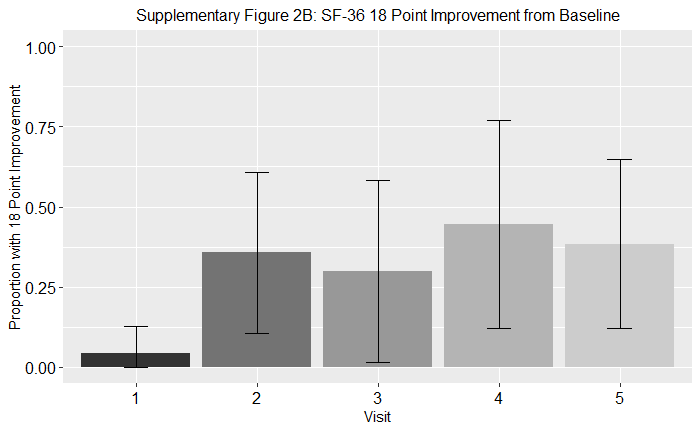

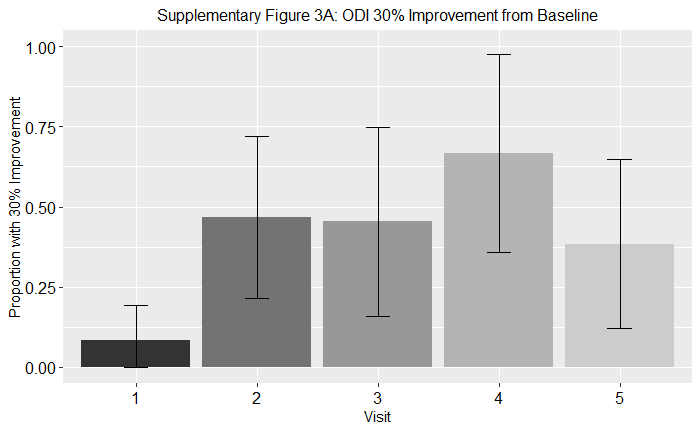

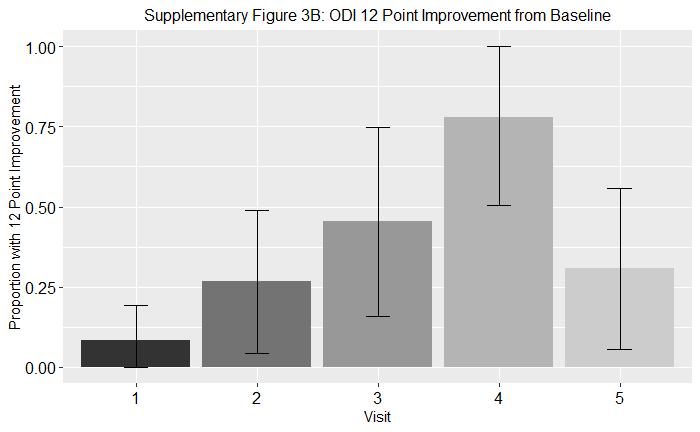


Denominator N = 23 14 10 9 13

Denominator N = 23 14 10 9 13

Denominator N = 24 15 11 9 13

Denominator N = 24 15 11 9 13

Supplement: Supplementary file 1 — Additional file 1: Figure S1. (A) Proportion and 95% confidence intervals of patients with Numeric Rating Scale (NRS) score improvements of at least 30%. Proportions at each post-injection time point were 17.2, 54.2, 52.9, 41.2, and 44.4%, respectively. (B) Estimated NRS MCID of 2 points for patients reporting at least 30% improvement in pain scores. Proportions at each post-injection time point were 17.2, 45.8, 47.1, 35.3, and 38.9%, respectively. Visits are defined as follows: visit 1 (2 wks), visit 2 (6–8 wks), visit 3 (12 wks), visit 4 (6 mo), visit 5 (≥ 1 yr). The number of patients (N) with follow up data is listed below visit number. Figure S2. (A) Proportion and 95% confidence intervals of patients with Short Form-36 Health Survey (SF-36) score improvements of at least 30%. Proportions at each post-injection time point were 4.4, 35.7, 30.0, 44.4, and 30.8%, respectively. (B) Estimated SF-36 MCID of 18 points for patients reporting at least 30% improvement in pain scores. Proportions at each post-injection time point were 4.4, 35.7, 30.0, 44.4, and 38.5%, respectively. Visits are defined as follows: visit 1 (2 wks), visit 2 (6–8 wks), visit 3 (12 wks), visit 4 (6 mo), visit 5 (≥1 yr). The number of patients (N) with follow up data is listed below visit number. Figure S3. (A) Proportion and 95% confidence intervals of patients with Oswestry Low Back Pain Disability Index (ODI) score improvements of at least 30%. Proportions at each post-injection time point were 8.3, 46.7, 45.5, 66.7, and 38.5%, respectively. (B) Estimated ODI MCID of 12 points for patients reporting at least 30% improvement in pain scores. Proportions at each post-injection time point were 8.3, 26.7, 45.5, 77.8, and 30.8%, respectively. Visits are defined as follows: visit 1 (2 wks), visit 2 (6–8 wks), visit 3 (12 wks), visit 4 (6 mo), visit 5 (≥1 yr). The number of patients (N) with follow up data is listed below visit number [file 12891_2020_3126_MOESM1_ESM.docx]
